# Hypoglycemia in patients with congenital muscle disease

**DOI:** 10.1186/s12887-020-1909-5

**Published:** 2020-02-06

**Authors:** Leslie H. Hayes, Pomi Yun, Payam Mohassel, Gina Norato, Sandra Donkervoort, Meganne E. Leach, Rachel Alvarez, Anne Rutkowski, Natalie D. Shaw, A. Reghan Foley, Carsten G. Bönnemann

**Affiliations:** 1 Neuromuscular and Neurogenetic Disorders of Childhood Section, Neurogenetics Branch, National Institute of Neurological Disorders and Stroke, National Institutes of Health, Building 10, Room 2B 39, MSC 1477, 10 Center Drive, Bethesda, MD 20892 USA; 2 Department of Neurology, Boston Children’s Hospital, 300 Longwood Ave, Boston Children’s Hospital, Fegan 11, Boston, MA 02115 USA; 3 Office of Biostatistics, National Institute of Neurological Disorders and Stroke, National Institutes of Health, Building 10, Room 2A 23, 10 Center Drive, Bethesda, MD 20814 USA; 4 Division of Neurology, Oregon Health and Science University, 3181 SW Sam Jackson Park Rd, Portland, OR 97239 USA; 5 Congenital Muscle Disease International Registry, Los Angeles, California USA; 6 Cure CMD, 19401 S. Vermont Avenue, Suite J100, Torrance, Los Angeles, CA 90502 USA; 7 Kaiser Southern California Permanente Medical Group, Los Angeles, California USA; 8 Pediatric Neuroendocrinology Group, Clinical Research Branch, National Institute of Environmental Health Sciences, Research Triangle Park, Durham, NC 27709 USA

**Keywords:** Hypoglycemia, Ketotic hypoglycemia, Congenital muscular dystrophies, Neuromuscular

## Abstract

**Background:**

Only a few small studies have previously reported episodes of hypoglycemia in children with neuromuscular diseases; however, there has been no broader investigation into the occurrence of hypoglycemia in children with congenital muscle disease (CMD).

**Methods:**

Pediatric patients enrolled in the CMD International Registry (CMDIR) with a history of hypoglycemia were included in this retrospective review. Hypoglycemic episodes and associated clinical and biochemical characteristics were characterized.

**Results:**

Ten patients with CMD (5 with *LAMA2*-related muscular dystrophy) reported at least one episode of hypoglycemia beginning at an average age of 3.5 years. Predominant symptoms included altered mental status and nausea/vomiting, and laboratory studies demonstrated metabolic acidosis and ketonuria, consistent with ketotic hypoglycemia.

**Conclusion:**

Patients with CMD may have an increased risk of hypoglycemia during fasting, illness, or stress due to their relatively low muscle mass and hence, paucity of gluconeogenic substrate. Clinicians should therefore maintain a high index of suspicion for hypoglycemia in this high-risk patient population and caregivers should routinely be trained to recognize and treat hypoglycemia.

## Background

Hypoglycemia, defined as a blood glucose level below 60 mg/dl (3.3 mmol/L) in infants and children [1], has been reported sporadically in children with neuromuscular disease [2, 3]. Bruce et al. described two girls with spinal muscular atrophy type II (muscle mass only 10% of body weight) with recurrent, severe hypoglycemic episodes that went unrecognized until the girls became comatose and were found to have blood glucose levels of 30–34 mg/dl (1.7–1.9 mmol/L) and metabolic acidosis [2]. In one girl (case #2), hypoglycemia frequently developed in the morning after fasting overnight [2]. Shu et al. reported a similar case; a 7-year-old boy with congenital muscular dystrophy with hypoglycemic episodes both upon awakening and during the course of viral illnesses [3]. A 34-h fasting study in 9 boys with Duchenne muscular dystrophy did not precipitate hypoglycemia, however these patients demonstrated lower alanine levels [4], suggesting a decreased reserve of gluconeogenic substrate from muscle. Reports of hypoglycemia are equally rare among adults with neuromuscular disease [5, 6]. Importantly, the incidence of hypoglycemia among adults and children with neuromuscular disease has not been systematically investigated.

It has been proposed that low muscle mass in the context of muscle atrophy predisposes children with neuromuscular disorders to hypoglycemia. In healthy children, glucose levels can be maintained by hepatic glycogenolysis during periods of fasting for approximately 8–12 h [7]. As glycogen stores are depleted, however, amino acids derived from the breakdown of muscle tissue become essential substrates for hepatic gluconeogenesis [7]. In the glucose-alanine cycle, for example, alanine is released into the bloodstream by skeletal muscle and taken up by the liver [7]. There, it is converted to pyruvate which then enters the gluconeogenic pathway, creating glucose that can be shuttled back to muscle as an energy source [7]. Thus, when a patient with low muscle mass fasts or has increased energy requirements (e.g. during illness), he/she may be at risk for developing hypoglycemia due to inadequate gluconeogenic substrate from muscle. This risk is compounded in children because of their limited hepatic glycogen stores compared with adults [8, 9].

In this retrospective study, we identified 10 patients with a confirmed molecular or clinical and immunohistological diagnosis of congenital muscular dystrophy or congenital myopathy who experienced at least one episode of documented hypoglycemia. We report the frequency, common triggers, and symptoms associated with hypoglycemia episodes as well as any accompanying biochemical abnormalities available. We suggest that these patients deserve prompt evaluation of glucose levels when presenting with signs and symptoms of hypoglycemia and routine evaluation of glucose homeostasis.

## Methods

An IRB-approved study announcement was distributed electronically to all members of the Congenital Muscle Disease International Registry (CMDIR) by CMDIR staff. Families who expressed interest in participating in this study were then directed to contact the investigator (LH) via e-mail and medical information release consents were secured to request and review participants’ medical records. Patients were included if they: 1) had an established clinical and molecular diagnosis of congenital muscular dystrophy or congenital myopathy (based on genetic testing and/or muscle biopsy), and 2) had a history of at least one documented episode of symptomatic hypoglycemia (defined as a blood glucose level < 60 mg/dL) between the ages of 1–10 years.

**Fig. 1:**
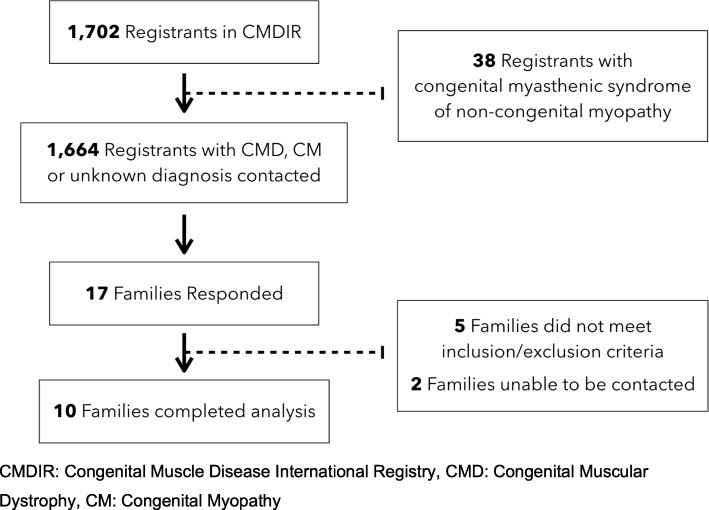
Study Recruitment Flow. CMDIR: Congenital Muscle Disease International Registry, CMD: Congenital Muscular Dystrophy, CM: Congenital Myopathy

Of the 1702 patients registered in the CMDIR, 38 registrants had an established diagnosis of a congenital myasthenic syndrome and were not included. The remaining 1664 patients were contacted, and 17 families responded to the inquiry and were then contacted directly by the investigators. Of the 17 patients with congenital muscle disease, five did not meet inclusion/exclusion criteria because they reported isolated neonatal hypoglycemia or could not provide documentation of hypoglycemia. Two patients did not respond to further emails from the study staff. The remaining 10 patients were included in the review of records and were contacted for necessary clarifications. A flowchart of study recruitment for patients is shown in Fig. 1.

Anthropometric data was also obtained from age-, sex-, and BMI (body mass index)-matched patients with congenital muscular dystrophy *without* a history of hypoglycemia who were evaluated at the National Institutes of Health (NIH) as part of an IRB-approved natural history study (protocol 12-N-0095). Note that in patients with congenital muscle disease, as well as in other patients with joint contractures, standing height is approximated using ulnar length [10].

## Results

### Patient characteristics

The 10 patients (6 male, 4 female) were all confirmed to have congenital muscle disease. Seven patients had congenital muscular dystrophies: *LAMA2-*related dystrophy (*n* = 5), *COL6-*related dystrophies (*n* = 1) or α-dystroglycanopathy (*n* = 1), and three patients had congenital myopathies: *DNM2-*related myopathy (*n* = 1) and *TTN-*related myopathy (*n* = 2). Diagnoses were confirmed through review of genetic testing and/or muscle biopsy with immunohistological studies. The average age of the patient at the time of data review was 11 years. Nine out of 10 patients had additional medical records relevant to an episode of hypoglycemia, which included documentation of 32 independent hospital or emergency department visits. Additional patient characteristics are reported in Table 1.

**Table 1:** Cohort Characteristics

Patient Characteristics							Hypoglycemia-related Characteristics						
Patient ID	Age at Survey (y)	Age at Diagnosis (y)	Diagnosis		Ambulation status	FVC % pred (age)	Age of 1st episode of hypoglycemia (y)	Caregiver estimate of # of lifetime hypoglycemic episodes	# of hospital encounters reviewed	Age of most recent hypoglycemic episode (y)	History of FTT	Supplemental Nutrition	BMI percentile^b^
ID 1	7	0	*LAMA2*-RD	c.2 T > C; c.47delG & muscle biopsy	N	51% (5y)	5	5–8	6	6^a^	Y	–	74
ID 2	6	0	*LAMA2*-RD	c.7732 C > T & muscle biopsy	N	–	1	16–25	2	5^a^	Y	G-Tube, GJ, TPN	–
ID 3	14	1	*LAMA2*-RD	c.2538-1G > C & muscle biopsy	N	86% (13y)	2	> 25	7	14^a^	Y	G-tube	82
ID 4	14	2	*DNM2*-RM	c.1102G > A	Y	–	1	> 25	4	3	N	–	60
ID 5	5	2	*LAMA2*-RD	c.3976C > T; c.4523 + 1G > A & muscle biopsy	N	–	4	< 5	4	5^a^	–	–	–
ID 6	14	1	*TTN*-RM/ CNM	c.32854 G > C; c.37112-1G > A & muscle biopsy	Y	44% (12y)	2	< 5	2	7	Y	G-tube	< 0.1
ID 7	7	0	*TTN*-RM	c.92992A > G; c.19241C > A & muscle biopsy	Y	104% (8y)	2	5–8	5	5	Y	G-tube	48
ID 8	9	0	*LAMA2*-RD	c.725G > A; c.7572 + 1G > T & muscle biopsy	N	–	5	5–8	1	8	N	G-tube	–
ID 9	17	1	*COL6*-RD	COL6A3i15/e16;c.6157–9 _c.6177del30 & muscle biopsy	N	26% (16y)	7	< 5	1	8	Y	–	0.2
ID 10	10	10	aDG	muscle biopsy	N	–	4	< 5	–	4	Y	G-tube	–

*ID* identification, *y* years, *Pred* Predicted, *FVC* Forced Vital Capacity on Pulmonary Function Testing, *BMI* body mass index, *RD* related dystrophy, *RM* related myopathy, *aDG* alpha-dystroglycanopathy, *Y* Yes, *N* No, *G-tube* gastrostomy tube, *GJ* gastro-jejunostomy tube, *TPN* total parenteral nutrition, ^a^Hypoglycemia episodes ongoing at the time of data collection, ^b^Reported closest available to onset of hypoglycemia, − Not available

### Characteristics of hypoglycemic episodes

The first episode of confirmed hypoglycemia occurred at an average age of 3.5 years (range 1.6–7 years). While most caregivers reported eight or fewer episodes of hypoglycemia, three caregivers reported more than 15 episodes of hypoglycemia (IDs 2,3,4; Table 1). Four of the five patients who were age 10 or older at the time of the study reported complete resolution of these episodes by 10 years of age.

All patients had symptoms of lethargy/sleepiness at the time of hypoglycemic episodes. Difficulty concentrating, decreased responsiveness, and nausea/vomiting were also commonly reported symptoms (Table 2), however gastrointestinal symptoms may have been secondary to a concurrent illness rather than hypoglycemia. Of note, there were no reports of changes in vision, seizures, tremors or nightmares. Eight out of the 10 families reported general illness as a trigger for a hypoglycemic episode. Skipping meals (*n* = 3), constipation (*n* = 1) and heavy exercise (*n* = 1) were reported as additional potential triggers for hypoglycemic episodes.

**Table 2:** Symptoms Reported

# Cases	Symptoms Reported
10	Lethargy/sleepiness
9	Decreased responsiveness
	Nausea/vomiting
7	Difficulty concentrating
6	Loss of coordination
	Sweating and/or flushed
5	Slurred speech
	Headache
	Fast or pounding heartbeat
3	Inconsolable crying
	Irritability
	Confusion
2	Tingling or numbness in the lips or tongue
	Anxiety
	Lightheadedness or dizziness
	Problems with memory
1	Erratic behavior
0	Nightmares
	Seizures
	Changes in vision
	Trembling

### Medical evaluation of hypoglycemia

**Fig. 2:**
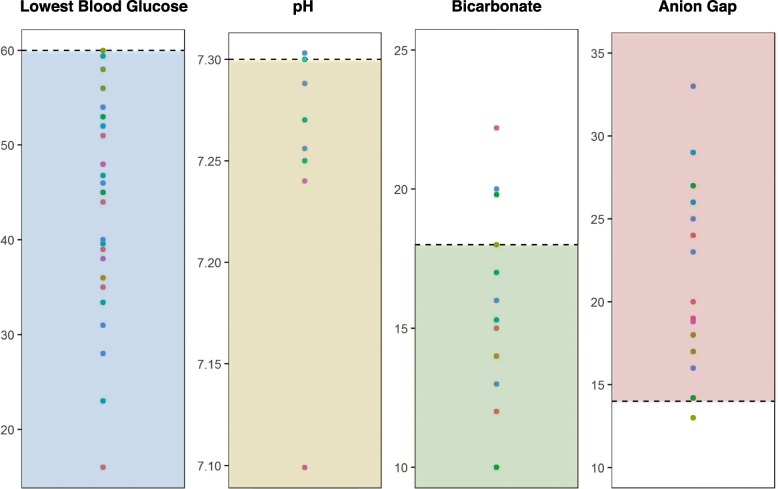
Laboratory Test: blood glucose (mg/dL), pH (units), bicarbonate (mmol/L), anion gap (mmol/L). All values were collected from hospital visits for hypoglycemia. Each point color corresponds with a single patient. Cutoffs (dotted line) of clinical significance for each lab test and concern ranges (shaded) suggestive of hypoglycemia for blood glucose values and metabolic acidosis for bicarbonate, anion gap and pH are shown

Nine patients had laboratory testing and hospital records available for review. Patients were evaluated for hypoglycemia in the emergency department or during an inpatient hospital admission an average of 4 times (ranging from 1 to 7) per patient. Blood glucose levels ranged from 16 to 60 mg/dl (average of 45 mg/dL)), and hypoglycemia was accompanied by increased anion gap (AG) metabolic acidosis (defined as pH < 7.3, bicarbonate < 18 mmol/L, and AG > 14 mmol/L) in all cases (Fig. 2). Urine samples were collected during eight encounters and demonstrated ketonuria in 87% of samples. Patients were initially treated with normal saline (NS) (45.5%), D5 ½NS (22.7%) or D10 ½NS (31.8%), suggesting delayed recognition of hypoglycemia by medical providers. Indeed, medical records indicated that on at least three separate occasions, the diagnosis of hypoglycemia was not initially considered despite the presence of vomiting, decreased oral intake, and/or lethargy.

### Home management of hypoglycemia

Seven families used a home glucometer to monitor blood glucose levels. Three families checked their child’s blood sugar regularly regardless of symptoms (e.g. three times a day or once in the middle of the night), while the four remaining families checked blood glucose only when their child became ill, had not eaten for a prolonged period of time, or showed symptoms of hypoglycemia. Furthermore, caregivers reported frequent blood glucose levels of 60–80 mg/dL with associated symptoms that would prompt them to intervene. In the home setting, hypoglycemia was treated with glucose-rich food (*n* = 6) or medication (*n* = 3; oral glucose tablets or gel), by initiating a feed via G-tube (*n* = 5), or by using a glucagon injection kit (*n* = 1). Two additional families had been prescribed a glucagon injection kit but had never used it. No parents reported treatment of hypoglycemia with glucocorticoids. Despite having access to one or more home remedies, most families reported that their child’s blood glucose did not normalize until dextrose containing IV fluids were administered.

### Anthropometric data

Anthropometric data was reviewed in patients with CMD with or without a history of hypoglycemia to investigate a potential causal link between low muscle mass and hypoglycemic episodes. In the absence of available data on body composition, BMI percentile was used as a proxy for muscle mass. However, there was no difference in BMI percentiles between our cohort and patients with CMD controls who have not reported hypoglycemia. Furthermore, this cohort had BMIs that spanned a large range of percentiles, based on CDC growth charts for healthy controls (Table 1).

## Discussion

In this retrospective registry-based study, we report a cohort of 10 patients with genetically and/or pathologically confirmed forms of congenital muscle disease (congenital muscular dystrophies or congenital myopathies) who experienced at least one episode of hypoglycemia. We show that these hypoglycemic episodes are typically associated with clinical symptoms, ranging from increased fatigue to decreased alertness and are often associated with profound metabolic abnormalities consistent with a ketotic hypoglycemic state. Consistent with previous reports, these hypoglycemic episodes are typically triggered by prolonged periods of fasting or situations of increased glucose demand such as intercurrent illness or increased physical activity. Hypoglycemic episodes were recurrent and required aggressive treatment in most of the patients studied in this cohort.

There are multiple potential mechanisms by which patients with congenital muscle disease may be at risk for developing hypoglycemia. Decreased muscle mass is believed to account for the higher risk of hypoglycemia in patients with neuromuscular disease [5, 6]. Indeed, skeletal muscle is an essential source of amino acid substrate for gluconeogenesis during periods of prolonged fasting. While we did not have access to urine creatinine or DEXA data to estimate lean muscle mass, we did not find a strong association between hypoglycemia and BMI percentile. Further, as the majority of hypoglycemic episodes occurred in patients younger than 10 years-of-age and resolved over time, we suspect that the pathophysiology of hypoglycemia in CMD patients may overlap with that of idiopathic ketotic hypoglycemia in normal children [11].

There may also be disease-specific factors that predispose CMD patients to ketotic hypoglycemia. For example, *LAMA2-*related dystrophy is caused by a deficiency of laminin-211 (also known as merosin). It is of interest that laminin-211 is expressed not only in muscle tissue but also in the basement membrane of pancreatic insulin-producing beta cells [12, 13]. However, hyperinsulinism is typically associated with non-ketotic hypoglycemia and the majority of CMD patients had ketonuria. Another possibility is that the increased metabolic rate in pediatric patients with congenital muscle disease is contributory, as has been described in patients with Duchenne muscular dystrophy [14]. Future prospective studies in a larger patient cohort that include detailed biochemical parameters will be necessary to better understand the cause of hypoglycemia among patients with CMD.

The recruitment of patients to this study was limited by family initiative and engagement with the CMDIR and hence resulted in a low response rate. Since the small sample size and retrospective nature limits our conclusions, it will be necessary to further investigate the occurrence of hypoglycemia in patients with congenital muscle disease in a prospective study.

In conclusion, we report hypoglycemia as an important clinical occurrence in a cohort of 10 pediatric patients with congenital onset muscle disease. These patients appear to be more susceptible to hypoglycemia during early childhood, possibly due to low muscle mass resulting in inadequate gluconeogenic substrate. Thus, a glucose level should be checked in any patient who presents with an unexplained change in level of alertness, nausea and/or vomiting Specifically we recommend that (1) patients and their caregivers should monitor glucose levels during prescribed fasts (in preparation for surgery or other procedures requiring anesthesia) or during periods of illness when normal oral intake cannot be maintained; (2) first line providers should be aware of an apparent increased risk of hypoglycemic episodes in pediatric patients with congenital muscle disease and tailor diagnostic testing and fluid resuscitation protocols accordingly; and (3) providers should consider screening CMD patients for hypoglycemia with fasting glucose levels to identify those at risk.

## Conclusions

Children with congenital muscle disease are at risk for episodes of ketotic hypoglycemia, often triggered by illness or fasting, which we postulate is related to low muscle mass and possible intrinsic muscle factors. Clinically, patients should be monitored closely for symptoms of hypoglycemia, be promptly evaluated and treated, and preventative measures should be considered.

## Data Availability

The datasets used and/or analyzed during the current study are available from the corresponding author on reasonable request.

## Abbreviations

BMI: Body mass index
CMD: Congenital muscle disease
CMDIR: Congenital Muscle Disease International Registry
DMD: Duchenne muscular dystrophy
N: Number
NIH: National Institutes of Health
SMA: Spinal muscular atrophy
